# The Barcode of Life Data Portal: Bridging the Biodiversity Informatics Divide for DNA Barcoding

**DOI:** 10.1371/journal.pone.0014689

**Published:** 2011-07-27

**Authors:** Indra Neil Sarkar, Michael Trizna

**Affiliations:** 1 Center for Clinical and Translational Science, University of Vermont, Burlington, Vermont, United States of America; 2 Department of Microbiology and Molecular Genetics, University of Vermont, Burlington, Vermont, United States of America; 3 Department of Computer Science, University of Vermont, Burlington, Vermont, United States of America; 4 Consortium for the Barcode of Life, Smithsonian Institution, Washington D.C., United States of America; King's College London, United Kingdom

## Abstract

With the volume of molecular sequence data that is systematically being generated globally, there is a need for centralized resources for data exploration and analytics. DNA Barcode initiatives are on track to generate a compendium of molecular sequence–based signatures for identifying animals and plants. To date, the range of available data exploration and analytic tools to explore these data have only been available in a boutique form—often representing a frustrating hurdle for many researchers that may not necessarily have resources to install or implement algorithms described by the analytic community. The Barcode of Life Data Portal (BDP) is a first step towards integrating the latest biodiversity informatics innovations with molecular sequence data from DNA barcoding. Through establishment of community driven standards, based on discussion with the Data Analysis Working Group (DAWG) of the Consortium for the Barcode of Life (CBOL), the BDP provides an infrastructure for incorporation of existing and next-generation DNA barcode analytic applications in an open forum.

## Introduction

Computational approaches have increasingly become a keystone element for the advancement of biological science [1], [2]. This has especially been the case in light of significant advances in sequencing technologies, such as those employed by major initiatives like DNA Barcoding [3], [4]. The basic premise of DNA Barcoding is to make use of defined segments of molecular sequence data for robust identification of life on Earth. DNA Barcoding has emerged as a systematic framework for the identification of fauna [4], [5] (and, more recently, flora [6]) according to a standardized reference library of molecular sequences [7], [8]. A number of tools and approaches have been developed for the analysis of DNA Barcode data [9], [10], [11], [12], , including those associated with classification of previously un-identified sequences. Collectively, the suite of analytic tasks in DNA Barcoding involves a wide range of analytic procedures (from taxon identification to data visualization to linkage with complementary knowledge sources). However, a limiting step for the community at large has been the limited availability of these innovative approaches.

To meet the ultimate goal of developing robust and efficient mechanisms for identification of biota using fragments of molecular sequence, the DNA Barcoding community will depend on a reliable framework for leveraging the latest innovations in classification approaches. In advance of approaches and classification systems being incorporated into production level interfaces (such as the Barcode of Life Data System [BOLD; http://www.boldsystems.org]), there may be great benefit for researchers to explore a range of computational approaches that are still in an “experimental” phase.

Service oriented workflow management and analysis toolkits (e.g., Taverna [14] or CIPRES [http://www.phylo.org/]) have been developed that cater to a range of communities. These systems are pioneering a new set of computational approaches that enable integration of analytic approaches across the Internet through the use of “Web services.” Amidst these significant innovations, there remain many tools that may be of interest for researchers but are not accessible via Web services. The phylogenetic community has seen great benefit in integration of common tools and techniques into approachable applications (e.g., MEGA [15]). Within the DNA Barcoding community, the vast majority of analytic tools that are not available from BOLD are only available in “stand-alone” form. Further reducing their exploration by the DNA Barcoding community are the set of pre-requisites that are often needed.

Since the early days of DNA Barcoding, the Data Analysis Working Group (DAWG) of the Consortium for the Barcode of Life (CBOL) has been comprised of a heterogeneous mix of computer scientists, mathematicians, and biologists (both computational and traditional). Collectively, this group has developed a wide array of analytic approaches that are specifically designed for exploring DNA Barcode data. Initially focused on the development of data analytics in support of DNA Barcode based identification, DAWG is concerned with analytic approaches that reflect the full complement of DNA sequence analysis (e.g., identification, clustering, benchmarking, visualization, and developing simulated data sets). However, the heterogeneous group of members also presented the challenge of developing a heterogeneous set of pre-requisites and interfaces for their resulting applications. DAWG thus prioritized the development of a common framework to share the rich array of methods developed by DAWG members. The resulting Barcode of Life Data Portal (BDP; http://bol.uvm.edu/) thus aims to bridge the divide between computational scientists and DNA Barcoding researchers. In this paper, we provide a brief description of the infrastructural steps required to instantiate the BDP and report on the current status of tool incorporation (which is mostly focused on the identification task, but the infrastructure is designed to accommodate the full spectrum of data analytic methods developed for DNA Barcode analysis).

## Results

The Barcode of Life Data Portal (BDP) was done in two phases. First, standardized input and output file formats were defined based on common requirements across the range of tools developed by DAWG members. Next, computational methods developed by DAWG members were incorporated into a common Web interface, which also provided links to common data sources. The resulting interface enables users to analyze the full range of publicly accessible DNA Barcode sequences as well as explore personal data sets using a growing number of methods being developed by DAWG.

### Common File Format Standards

**Figure 1:**
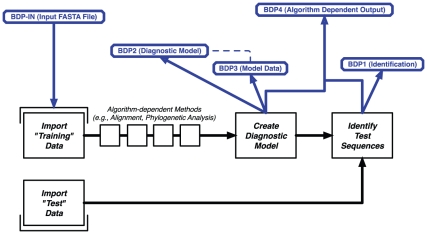
Overview of Common Data Analytic Process. Based on discussion with DAWG membership, the set of common steps (shown in black) that transcend across the range of data analytic applications was used to inform decisions for common file formats (shown in blue).

Through a meeting of DAWG members, a common workflow for the majority of DNA barcode analyses was developed by consensus (Figure 1). In summary, it was agreed that the DNA Barcode analysis process consists of a training set that is used to develop a classification model that is then used to classify new (test) data. Admittedly, the content of the training and test dataset can vary according to each algorithm. Nonetheless, the consensus was that this general workflow could be generalized for all of the tools that have been developed by DAWG to date.

Minimally, it was determined that all tools had some input file (BDP-IN) and would generate a set of predicted classifications (BDP1) for a set of test sequences. Depending on the algorithm, a diagnostic model (BDP2), model data (BDP3), and algorithm-dependent output files (BDP4) could also be generated. For consistency, all output files (BDP1-4) are required as output for a given classification operation. If an algorithm does not by default generate a BDP2, BDP3, or BDP4 file, the respective file simply contains the phrase “—NOT APPLICABLE—.” Further details about the file formats and there specifications can be seen here: http://bol.uvm.edu/docs/bdp_standards.pdf.

### Incorporation of Tools and Algorithms into the BDP

Priority is given to installation of tools and algorithms that are comprised of open-source and freely available pre-requisites. Understanding that there may be instances where commercial applications are required, the responsibility for acquiring and maintaining licenses for installation remain that of the tool developer. To date, none of the DAWG applications have specific requirements of commercial software. In cases where algorithms may have initially been developed using commercial applications, developers migrate to open-source equivalents before integration into BDP.

In addition to algorithms used for DNA Barcode analyses, BDP provides a mechanism for collecting and developing data sets. This system, called the “Aggregator” enables one to retrieve publicly accessible records from BOLD as well as those that have been given the “BARCODE” keyword flag in the INSDC databases (GenBank, EMBL, and DDBJ). Furthermore, the Aggregator enables one to submit additional sequences as “private” sequences that are formatted in the FASTA file format. As of December 2010, there are more than a half of a million DNA Barcode records in the Aggregator, which consist of almost 300,000 INSDC BARCODE keyword, more than 200,000 BOLD public records, and 21,000 private records. Private records are only accessible to the users that have uploaded them.

Publicly accessible records can be browsed and selected through a taxonomy browser (which is based on NCBI Taxonomy). Customized data sets can then be subjected to any of the analytic methods installed on BDP. Particular effort was made to ensure that the workflow, regardless of which algorithm is used, follows the common template. This was enabled strongly because of the enforcement of the common data formats as mentioned earlier. Step-by-step tutorials for how to create data sets, as well as how to run data through analyses, are available at the BDP Website (http://bol.uvm.edu/about.php). These tutorials also include screen shots of the major features of the BDP.

BDP utilizes a private log-in system to facilitate private barcode analysis, which is a strongly desired featured for researchers working with data that have not yet been published. Users can keep their barcode records and analytical results private until they are ready to publish. In addition, logging in enables users to form personal datasets and access previously completed analysis results. Although logging in is required to work with personal barcodes and datasets, non-registered users can still search the public Aggregator records and run any of the public datasets through BDP's analytical tools.

### Example Use of BDP: Preparing Data Release Report for Publication

In addition to classification studies, where the goal is to classify unknown sequences according to a reference library, another important goal of the BDP is to facilitate the data generation and reporting processes required for publication and public dissemination of DNA Barcode data. For example, as increasing DNA Barcode data are made available for publication in journals like PLoS ONE, an important aspect will be the development of standardized reports. In particular, a key set of metrics is required for publication (see: http://bol.uvm.edu/docs/data_release_guidelines.pdf). In a recent study involving Anopheles (Mosquito) data, 1770 records data were imported as “personal records” (http://bol.uvm.edu/add_personal_bold.php). These records originated from the BOLD system and labeled as “Anopheles” data within the private data set. Within the BDP, a validation step was performed using the BLAST algorithm to compare the sequences against known sequences associated with mosquitoes. The final analysis of these data within the BDP resulted in a PLoS ONE Barcode Data Release Report that could be used for final publication. In contrast to having to develop processes or use a myriad of biological sequence analysis tools, the researchers for this study were able to perform all the required tasks (import data, perform quality control, and do dataset analysis for publication) within the BDP.

## Discussion

The rapid maturation of DNA Barcoding since its inception in the early 2000's has resulted in a new era of species identification science [7]. DNA Barcoding is not meant in any way to replace or even replicate taxonomic studies [16], [17]; however, the advent of a short molecular sequence that can be used for robust and efficient identification may be an essential element in securing the future of developing catalogues of our planet's biota [18]. In support of the overall DNA Barcoding momentum, both INSDC interfaces (e.g., GenBank) and DNA Barcoding specific interfaces (e.g., BOLD) will be essential for providing centralized repositories with established standard approaches for data sequence searching and species identification. Nonetheless, there will be a continual need for common resources that enable researchers to explore the range of approaches that may be essential for providing a deeper understanding of DNA Barcode composition or empirical assessment of different classification approaches. The BDP strives to serve as a virtual “sandbox” for researchers to explore such applications through a common interface.

It is fully expected that researchers new to barcoding will make use of the central resource for sharing DNA Barcode data, BOLD. The BDP is designed as a complementary resource, which accesses BOLD data as well as data from other public resources like GenBank. However, in contrast to BOLD, which provides access to a limited number of algorithms for identification and visualization, BDP provides the potential to use any publicly available approach for identification and visualization. The current suite of approaches for identification are those that have been the focus of discussions within DAWG; however, it is fully anticipated that any researcher with a novel approach for identification could make their approach available for use via BDP. BOLD is thus analogous to how core data collection resources, such as GenBank, provide a primary forms for access and search of the data (e.g., searching through GenBank records using BLAST). BDP is then analogous to secondary exploration or identification of data as can be done with GenBank data through popular interfaces such as Ensembl Genome Browser. In contrast to both GenBank and Ensembl, BOLD and BDP are specifically focused on DNA barcoding applications. It is important to emphasize that BDP is not intended to be a replacement for BOLD, but is instead an environment where one can explore potentially “hot-off-the-press” approaches for exploring DNA Barcode data. In this way, BOLD and BDP together work synergistically to provide the DNA Barcode community with robust cataloguing and access to DNA Barcode data (BOLD) and the ability to explore these data using a wide array of available approaches (BDP).

Beyond DNA Barcoding, there may be great benefit in demystifying much of the statistical, computer science, and informatics approaches for biologists. One of the major goals of initiatives like the BDP is to thus bring biologists closer to the laboratory environment where new algorithms are devised. To this end, a major goal of DAWG is to increase the impact of experimental algorithms in the context of real data. As noted earlier, the BDP does not take on the responsibility for evaluating the tools and algorithms as they are incorporated. There are plans to eventually require that BDP tool contributors also provide benchmark datasets that are associated with published results.

The existing suite of tools incorporated into BDP consists of mostly sequence identification routines. This is not to suggest that the only emphasis in DNA Barcoding is developing sequence identification approaches but, admittedly, does reflect the majority of emphasis by the DAWG community since its inception. Nonetheless, DNA Barcoding will require a more comprehensive suite of tools for data analysis (including, but not limited to: phylogenetic, evolutionary analysis, information content, and sequence clustering). To this end, there may also be important tasks that need to be done for DNA Barcode data that can be facilitated using routines implemented in the BDP. For example, the “Barcode Data Release Report” tool implemented on BDP enables researchers to generate a report of the requisite statistical metrics required for publication. While many of these kinds of tools may eventually be incorporated in services such as offered by BOLD or GenBank, the volume of data that will become available in part due to major DNA Barcoding campaigns presents a current need for analyses to be done that can be met by services provided by BDP incorporated algorithms.

The standardization of file formats for enabling integration of tools into BDP establishes a robust infrastructure for subsequent incorporation of the next generation of analyses and data exploration tools that will be developed by the DAWG membership. The analytic emphasis of BDP, which is directly reflective of the relative expertise of DAWG, is primarily on sequence analysis and identification. Some approaches have been developed for linking to other data types (e.g., DNA Barcode Linker which leverages Google-based for a given sequence). To this end, the establishment of common file format standards increases the ability for anyone developing sequence analytic tools to contribute to the BDP, regardless of their membership in DAWG. The next major goal for the BDP is to complete incorporation of nearly a dozen algorithms and tools that have been developed by the DAWG since its beginnings (and thereby expand the analytic capabilities of BDP). While not planned for the immediate future, there may be great opportunities to also integrate other complementary analyses that can be done alongside DNA Barcode based data (e.g., geo-location or morphology based analyses).

Much of the emphasis to date within the DAWG community has been the development of approaches for sequence analysis for the purposes of data description and assessment of classification robustness. However, a major cadre of data analysis approaches has yet to be fully realized – data visualization. Accepting that the innovations in areas such as data visualization may likely come from outside the current DAWG community, the ability to develop tools that can be integrated into the BDP through the use of common standards becomes more important.

Currently, the BDP interface is set up to enable researchers to go through a given analytic operation in a linear fashion. There are plans that will enable researchers to combine the approaches from the available approaches within BDP. For example, one might want to start with a character-based analytic approach, and then depending on the results, move over into a more statistical model approach. The standardization of input and output formats facilitates the ability for researchers to go between algorithms more fluidly.

Interaction with the BDP is done exclusively through the PHP-based Web portal. The PHP interface is only one representation of the how one may be able to interact with BDP. The architecture of the BDP was designed to eventually enable REpresentational State Transfer (REST) based Web service access. There are plans to incrementally make various REST Web services available to the public, thus making possible the development of systems that leverage the BDP resources. These Web services will enable batch computing and facilitate tighter integration with other DNA Barcoding websites.

The Barcode of Life Data Portal (BDP) represents a first step towards bridging the divide between computational innovations in data analysis and meeting the needs of biologist end users. Through a Web based interface, researchers are able to aggregate sequences across public and personal data sources and then run them through a growing array of tools that are contributed by the DNA Barcode and sequence analysis communities. As a complement to resources such as GenBank and BOLD, BDP enables the ability for researchers to explore data with the latest community driven innovations in data analytics.

## Materials and Methods

The BDP was completely developed using the PHP language, and is thus developed as a Web-based, operating system independent environment. As such, with the exception of having a contemporary Web browser, there are no local software requirements for use. Registration is free and required to enable the tracking of jobs through the system. Housed at the Vermont Advanced Computing Center at the University of Vermont (http://www.uvm.edu/~vacc/), the BDP can potentially leverage the 7.1-teraflop IBM-based high-performance computer cluster (“Bluemoon”) for intensive operations. The vast majority of tools do not require sustained high-performance computing to carry out the bulk of operations; however, the ability to leverage Bluemoon facilitates those tasks that are amenable to parallel processing. This architecture also enables the ability to handle large numbers of simultaneous queries without performance impact on any given user.

In addition to the front-end interface that was developed in PHP, a number of supporting applications were required to support the range of algorithms that will eventually become part of the BDP. While commonplace for many in the informatics community, the installation of these applications can range in difficulty – from installation from source code to “simple” installation routines. Configuring individual applications to meet the requirements for use of a given tool can also further complicate the installation of a given application. As additional tools are incorporated in the BDP, DAWG works with the algorithm developer to install the necessary pre-requisites as well as integrate it into the common workflow (as shown in Figure 1). The BDP serves as a community portal for computational innovations in data analytics and reporting; it does not explicitly evaluate the performance of any given tool against others. As of this writing, there are five tools available in BDP, as shown in Table 1.

**Table 1:** List of current tools available in the BDP.

Tool Name	Category	Publication	Description
BLOG (Barcoding with LOGic formulas)	Identification	Bertolazzi P, Felici G, Weitschek E. Learning to classify species with barcodes. BMC Bioinformatics. 2009 Nov 10; 10 (Suppl 14): S7	BLOG (Barcoding with LOGic formulas) is a character-based identification tool based on Logic Mining techniques. The identification process is comprised of two steps. The first step is feature selection, where the problem of selecting a small number of relevant features if formulated as an integer programming problem. The second step is the identification of the logic formulas that separate each class from all the others. This task is accomplished using the *Lsquare* system for logic mining.
DNA Barcode Linker	Identification	Hajibabaei M, Singer G. Googling DNA sequences on the World Wide Web. BMC Bioinformatics. 2009 Nov 10; 10 (Suppl 14): S4	This is an interface to the DNA Barcode Linker website hosted by the Bioinformatics Laboratory at Concordia University, Montreal, Quebec. It was developed by Gregory Singer and Hamid Nikbakht, with technical assistance from Lee Zamparo.Searches of the DNA barcode library are based on an algorithm developed by Gregory Singer, Mehrdad Hajibabaei and Donal Hickey. This search algorithm is informally called GoogleGene.
BLAST	Identification	Camacho C, Coulouris G, Avagyan V, Ma N, Papadopoulos J, Bealer K, Madden T. Blast +: architecture and applications. BMC Bioinformatics. 2009 Dec 15; 10:421	BLAST stands for Basic Local Alignment Search Tool. It was originally designed at the NIH by Eugene Myers, Stephen Altschul, Warren Gish, David J. Lipman and Webb Miller in 1990. The Data Portal implements the current BLAST+ release 2.2.23 from March 2010. The algorithm emphasizes speed over sensitivity, so BLAST is not optimized for barcode identification, but still is able to provide valuable estimate identifications.
Barcode Data Release Report	Dataset Analysis	Guidelines to Authors of BARCODE Data Release Papers for Submission to PLoS ONE (http://www.barcoding.si.edu/guidelines.html)	This tool generates a report of all of the required statistical measures expected in a barcode data release report submitted to PLoS One.
Sequence Composition	Dataset Analysis	None	This tool gives a general statistical report of the nucleotide composition of the sequences in a dataset. Results are available for each sequence, as well as the dataset as a whole.
